# Dry eye syndrome and the subsequent risk of chronic fatigue syndrome—a prospective population-based study in Taiwan

**DOI:** 10.18632/oncotarget.25544

**Published:** 2018-07-17

**Authors:** Chih-Sheng Chen, Hui-Man Cheng, Hsuan-Ju Chen, Shin-Yi Tsai, Chia-Hung Kao, Hui-Ju Lin, Lei Wan, Tse-Yen Yang

**Affiliations:** ^1^ Department of Chinese Traumatology, Division of Chinese Traumatology Medicine, China Medical University Hospital, China Medical University, Taichung, Taiwan; ^2^ Department of Traditional Chinese Medicine, Asia University Hospital, Taichung, Taiwan; ^3^ Graduate Institute of Chinese Medicine, College of Chinese Medicine, China Medical University, Taichung, Taiwan; ^4^ Department of Integration of Traditional Chinese and Western Medicine, China Medical University Hospital, Taichung, Taiwan; ^5^ School of Chinese Medicine, College of Chinese Medicine, China Medical University, Taichung, Taiwan; ^6^ Management Office for Health Data, China Medical University Hospital, Taichung, Taiwan; ^7^ Department of Public Health, China Medical University, Taichung, Taiwan; ^8^ Asia University, Taichung, Taiwan; ^9^ Department of Laboratory Medicine, Mackay Memorial Hospital, Mackay Medical College, New Taipei City, Taiwan; ^10^ Department of Health Policy and Management, Bloomberg School of Public Health, Johns Hopkins University, Baltimore, Maryland, USA; ^11^ Graduate Institute of Clinical Medical Science and School of Medicine, College of Medicine, China Medical University, Taichung, Taiwan; ^12^ Department of Nuclear Medicine and PET Center, China Medical University Hospital, Taichung, Taiwan; ^13^ Department of Bioinformatics and Medical Engineering, Asia University, Taichung, Taiwan; ^14^ School of Chinese Medicine, China Medical University, Taichung, Taiwan; ^15^ Department of Ophthalmology, China Medical University Hospital, Taichung, Taiwan; ^16^ Department of Biotechnology, Asia University, Taichung, Taiwan; ^17^ Department of Obstetrics and Gynecology, China Medical University Hospital, Taichung, Taiwan; ^18^ Research Center for Chinese Medicine and Acupuncture, China Medical University, Taichung, Taiwan; ^19^ Molecular and Genomic Epidemiology Center, China Medical University Hospital, China Medical University, Taichung, Taiwan; ^20^ Department of Medical Research, China Medical University Hospital, China Medical University, Taichung, Taiwan; ^21^ Department of Medical Laboratory Science and Biotechnology, China Medical University, Taichung, Taiwan

**Keywords:** fatigue, dry eye syndrome, national health insurance research database (NHIRD), prospective cohort study

## Abstract

**Background and Aim:**

The clinical association between dry eye syndrome (DES) and chronic fatigue syndrome (CFS) remain unclear with less evidences. We aimed to investigate the relationship between CFS and DES using a national insurance and prospective cohort study.

**Methods:**

Data from the Longitudinal Health Insurance Database 2000 was applied to estimate the incidence of CFS among patients with DES, and their age- and sex-matched controls without DES over a long-term follow-up period. All participants were CFS free at baseline, before the interval (2005–2007), but were later diagnosed with CFS. DES patients and its relative matched controls were excluded prevalent CFS before the same interval.

**Results:**

We identified 884 patients with DES and 3,536 matched controls in baseline and estimated the hazard ratios for incident CFS in the follow-up period. Patients with DES had a 2.08-fold considerably increasing risk of developing CFS, compared to non-DES group. An elevated risk of developing CFS remained (1.61-fold risk) even after adjusting for age, sex, and comorbidities. There was a presence of increasing risk in DES-related CFS when CFS-related comorbidities existing (adjusted hazard ratio, 1.98, 95% confidence interval, 1.19–3.29; *p* < 0.01). The subsequent risk for CFS between DES and non-DES patients was significant increased with three or more annual medical visits, the adjusted risk for CFS was 4.88-fold risk (95% CI, 2.26–10.58, *p* < 0.001).

**Conclusion:**

We recommended that physicians should be aware of the increased risk of CFS among DES patients and adequately assess the health impacts among these patients.

## INTRODUCTION

Little is known about the risk factors influencing chronic fatigue syndrome (CFS) following dry eye syndrome (DES) [1–8]. A previous study has shown that most patients with DES were classified as having spectrum diseases, such as fibromyalgia (FM) [9], and that DES was related to FM, even among FM patients without comorbidities (1.40-fold risk) [9]. Furthermore, CFS has been shown to overlap with FM as well as other rheumatology diseases, such as Sjögren syndrome (SS) [10]. In addition, a previous clinical study has demonstrated that sicca symptoms existed in about 70% of CFS patients [11]. Furthermore, the recent study was shown that the SS patients were presence of dryness, pain, fatigue, even the disturbance of quality of life [12]. Another study showed that atopic syndromes were associated with the risk for CFS and the presence of multiple atopic syndromes was associated with a higher risk for CFS [13–15]. The disease burden of DES should be elevated the economic burden and personal health expenditure, especially in the DES treatment cost of Asian population [16].

The DES diagnostic process is usually conducted in clinics using the Schirmer’s strip test and/or the tear film break-up time [17], but this test was not properly assessed the DES impact. The DES management would be provided more solid evidence for confirmed diagnosis of DES as a surrogate marker of uncertainty diseases, like CFS, and well request DES assessment for the health insurance benefits as standard criteria. In addition, a recent study demonstrated that DES was significantly associated with antipsychotic drug use and self-reported symptoms of FM [15]. However, evidence-based clinical observations for clarified the relationship between DES and the subsequent risk of CFS still remain unclear. Moreover, no studies have used a prospective study to assess the risk of simple DES-related CFS in a population-based cohort, which randomly selecting from an insurance database covering 99% of residents in Taiwan [18].

The diagnostic criteria for CFS in Taiwan are based on the 1994 Fukuda definition [19, 20] and the 1998 United States Centers for Disease Control guidelines [20, 21]. However, since several diseases not classified as CFS can contribute to long-term, persistent fatigue [22–24], we aimed to investigate the relationship between DES and the subsequent risk of developing CFS as well as to clarify the relative risk of developing CFS adjusting for overlapped comorbidities by a population-based prospective cohort.

## RESULTS

**Figure 1:**
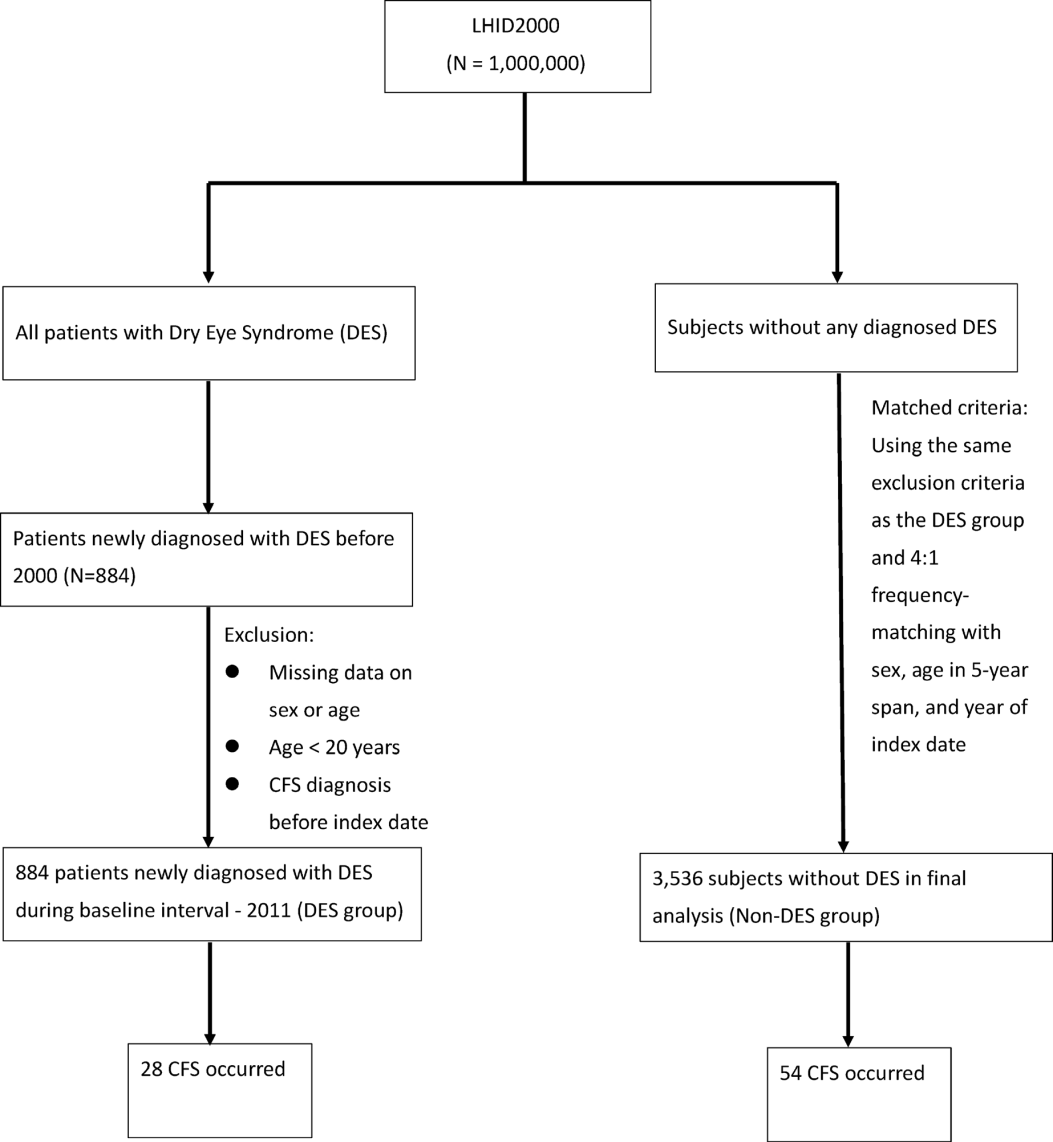
Study design for dry eye syndrome (DES) and subsequent chronic fatigue syndrome (CFS) risk

In the present study design (Figure 1), we aimed to mention the simple DES-related CFS risk in an Asian, health insurance database-based, population study using Taiwan National Health Insurance Research Database (NHIRD).

### Basic characteristics of the DES and non-DES groups

The basic characteristic of distribution among the variables was present that the age (continuous or category variables), gender and co-morbidities distributions. We identified 884 patients with DES and 3,536 age-, gender-matched controls. The baseline characteristics are shown in Table 1. There were no significant differences in the distributions of SLE or cancer between the DES and non-DES cohorts. However, significant differences were found for SS, rheumatoid arthritis (RA), depression, anxiety, sleep disturbance, irritable bowel syndrome (IBS), and FM between these two groups (Table 1).

**Table 1:** Demographic factors and comorbidity of study participants according to dry eye syndrome status

	No DES cohort *N* = 3,536		DES cohort *N* = 884		*p*-value
Variable	*n*	%	*n*	%	
Gender					0.99
Female	2616	73.98	654	73.98	
Male	920	26.02	230	26.02	
Age, years					0.99
20–64	2384	67.42	596	67.42	
≥ 65	1152	32.58	288	32.58	
Means (SD)	55.69	(16.25)	55.83	(16.16)	0.81
Comorbidity					
Cancers	108	3.05	31	3.51	0.56
SLE	7	0.20	4	0.45	0.33
SS	1	0.03	7	0.79	<0.001
RA	5	0.14	5	0.57	0.03
Depression	237	6.70	120	13.57	<0.001
Anxiety	556	15.72	260	29.41	<0.001
Sleep disturbance	1025	28.99	403	45.59	<0.001
IBS	262	7.41	134	15.16	<0.001
FM	972	27.49	337	38.12	<0.001

Abbreviation: DES, dry eye syndrome; SD, standard deviation; SLE, systemic lupus erythematosus; SS, Sjogren syndrome; RA, rheumatoid arthritis; IBS, irritable bowel syndrome; FM, Fibromyalgia.

### The risk of developing CFS among DES patients

In Table 2, the crude risks of developing CFS among DES patients were 2.08 (95% CI, 1.32–3.28, *p <* 0.01), and the risk of developing CFS after adjusting for age, gender, and comorbidities was 1.61 (95% CI, 1.01–2.58, *p <* 0.05). Whether the adjustment or not, the risks of CFS for women were 1.49 and 1.87-fold between the DES and non-DES cohorts, while the risks of CFS for men were 2.38 and 3.48-fold between the DES and non-DES cohorts. Moreover, in the analysis using age as a categorical variable (<65 and ≥65 aged groups), the risk for CFS was significantly higher (1.96–2.69) in <65 aged group. The ≥65 group had a slight risk of developing CFS (1.05–1.14) but this was not statistically significant. Interestingly, patients with any comorbidity (at least one) had a 1.98-fold higher risk of developing CFS. Patients with no comorbidities had a slightly elevated risk (aHR) of developing CFS (1.22-fold risk); however, this was not statistically significant. Another interesting part was about that the incidence of CFS among all patients (both those with and without DES) was about 2 per 1,000 person-years.

**Table 2:** Incidence density rates and hazard ratio for chronic fatigue syndrome according to dry eye syndrome status stratified by demographic factors and comorbidity

	Dry eye syndrome						Compared to no DES cohort			
	No			Yes			Crude HR (95% CI)		Adjusted HR (95% CI)	
Variables	Event	Person-year	IR	Event	Person-year	IR				
Overall	54	17986.70	3.00	28	4489.88	6.24	2.08	(1.32–3.28)^**^	1.61	(1.01–2.58)^*^
Gender										
Female	47	13400.12	3.51	22	3362.67	6.54	1.87	(1.13–3.10)^*^	1.49	(0.88–2.51)
Male	7	4586.58	1.53	6	1127.21	5.32	3.48	(1.17–10.36)^*^	2.38	(0.75–7.54)
Age, years										
20–64	33	12475.10	2.65	22	3098.20	7.10	2.69	(1.57–4.61)^***^	1.96	(1.12–3.42)^*^
≥ 65	21	5511.60	3.81	6	1391.68	4.31	1.14	(0.46–2.83)	1.05	(0.42–2.66)
Comorbidity^‡^										
No	17	8750.30	1.94	3	1329.56	2.26	1.16	(0.34–3.97)	1.22	(0.36–4.18)
Yes	37	9236.40	4.01	25	3160.32	7.91	1.97	(1.19–3.28)^**^	1.98	(1.19–3.29)^**^

Abbreviation: DES, dry eye syndrome; IR, incidence density rates, per 1,000 person-years; HR, hazard ratio; CI, confidence interval.

Adjusted HR: mutually adjusted for age, gender, and comorbidity in Cox proportional hazards regression.

^‡^ Patients with any one of cancers, systemic lupus erythematosus, rheumatoid arthritis, Sjogren syndrome, depression, anxiety, sleep disturbance, irritable bowel syndrome, and fibromyalgia were listed as the comorbidity group.

^*^ *p <* 0.05, ^**^ *p <* 0.01, ^***^ *p <* 0.001.

### The joint effect of DES and comorbidities

The adjusted risk of developing CFS among patients with DES and a related comorbidity was 3.77 (95% CI, 2.02–7.04, *p <* 0.001). In addition, the adjusted risk of developing CFS among patients without DES but who had any comorbidity was 1.89 (95% CI, 1.05–3.39, *p <* 0.05). However, the synergistic effect between DES and CFS was not significant (p for interaction = 0.44) in Table 3. The DES and its subsequently CFS risk might be independently existed association among the other overlapped symptoms.

**Table 3:** Joint effect between dry eye syndrome and comorbidity in association with chronic fatigue syndrome in study population

Variables		*N*	Event	Adjusted HR (95% CI)	p for interaction
DES	Comorbidity^‡^				0.44
No	No	1699	17	1.00	
No	Yes	1837	37	1.89 (1.05–3.39)^*^	
Yes	No	258	3	1.18 (0.35–4.04)	
Yes	Yes	626	25	3.77 (2.02–7.04)^***^	

Abbreviation: DES, dry eye syndrome; HR, hazard ratio; CI, confidence interval.

Adjusted HR: adjusted for gender and age in multiple logistic regression models.

^‡^ Patients with any one of cancers, systemic lupus erythematosus, rheumatoid arthritis, Sjogren syndrome, depression, anxiety, sleep disturbance, irritable bowel syndrome, and fibromyalgia were classified as the comorbidity group.

^*^ *p* < 0.05, ^***^ *p* < 0.001.

### Frequency of DES-related medical visits and the risk for CFS

We examined whether more frequent DES-related medical visits were associated with the risk of developing CFS. The results showed that having once to twice annual medical visit was associated with developing CFS (HR = 3.01, 95% CI, 1.61–5.64, *p <* 0.001) as was having three and more annual medical visits (HR = 4.88, 95% CI, 2.26–10.58, *p <* 0.001). In addition, the risk of developing CFS significantly correlated with frequency of medical visit numbers of DES (p for trend, *p <* 0.001, Table 4)

**Table 4:** Incidence density rates and hazard ratio for chronic fatigue syndrome risk stratified by the severity of dry eye syndrome

Average frequency for medical visit, per years	*N*	Event	Person-years	IR	Adjusted HR (95% CI)
No DES cohort	3536	54	17986.70	3.00	1.00
DES cohort					
<1	555	7	2975.44	2.35	0.63 (0.28–1.39)
1–2	244	13	1148.86	11.32	3.01 (1.61–5.64)^***^
≥3	85	8	365.57	21.88	4.88 (2.26–10.58)^***^
p for trend					<0.001

Abbreviation: IR, incidence density rates, per 1,000 person-years; HR, hazard ratio; CI, confidence interval; DES, dry eye syndrome.

Adjusted HR: adjusted for age, gender, and comorbidity in Cox proportional hazards regression.

^***^ *p* < 0.001.

### Cumulative incidence rate for CFS

**Figure 2:**
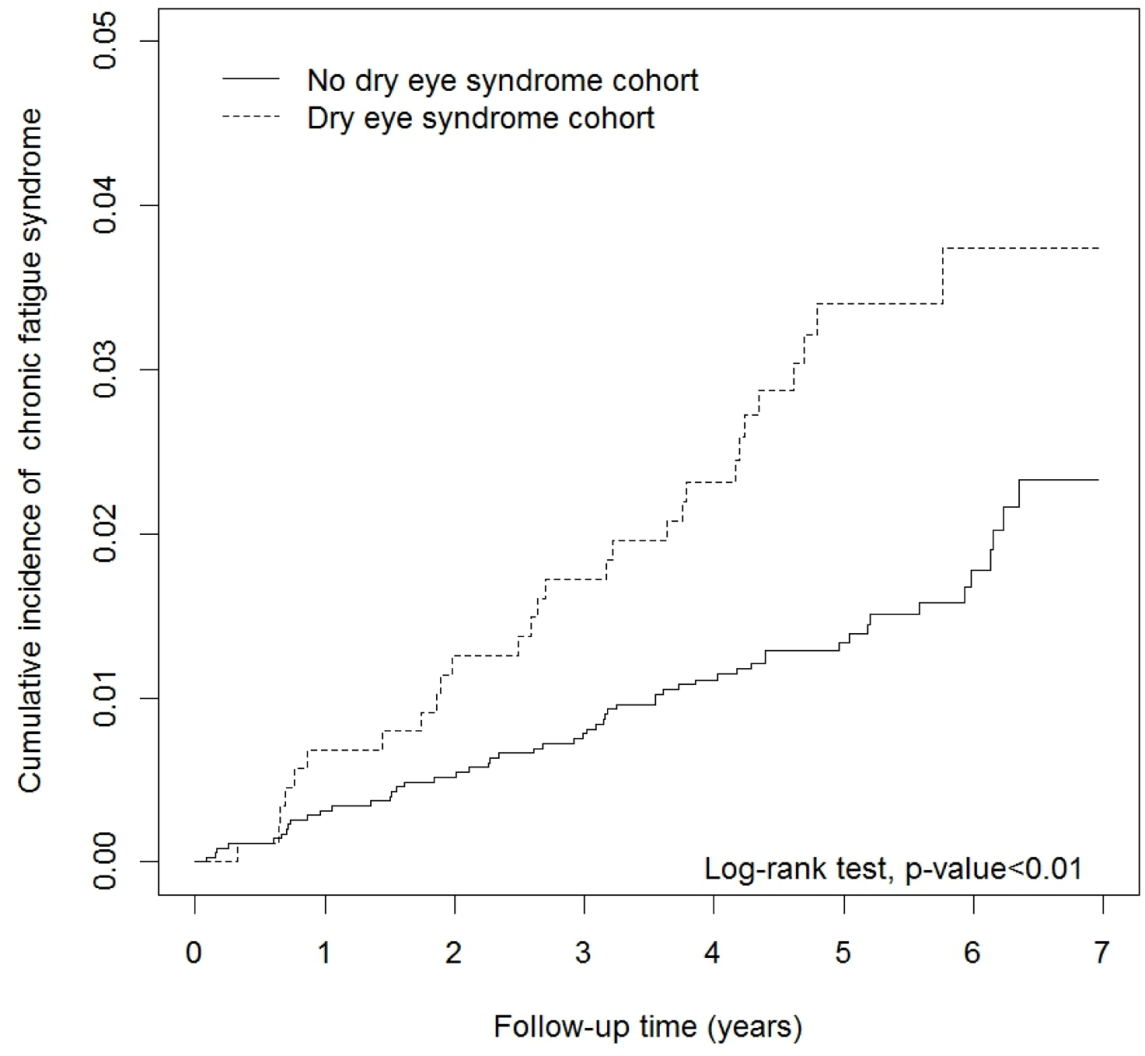
Cumulative incidence curves of chronic fatigue syndrome for dry eye syndrome (DES) and no DES groups

Cumulative incidence curves for CFS according to DES status are illustrated in Figure 2. The results of the log-rank test showed that the cumulative incidence rate of developing CFS was significantly higher in the DES group than in the non-DES group (*p <* 0.01 by the log-rank test). Furthermore, the cumulative incidence rate of developing CFS increased with follow-up time in both the DES and non-DES cohorts.

## DISCUSSION

CFS is a complex disorder and diagnosis is based on self-reported symptoms and the accompanying decrease in quality of life [29–32]. Physicians often classify disorders with long-term, persistent, and unreasonable fatigue as CFS. Furthermore, even if the resting period is continuous, fatigue and fatigue-related symptoms persist and are difficult to diagnose. Moreover, when faced with CFS, patients may be too overwhelmed to describe DES-related symptoms [33, 34]. In Taiwan, CFS is usually diagnosed using the criteria described above [35], not the Canadian criteria [36], nor the new criteria defined in an Institute of Medicine report [37, 38]. However, these criteria account for mental illnesses, meaning that the diagnosis of CFS rules out other potential causes, such as mental illness. In this study, we also tried to examine the risk of DES-related CFS as well as the CFS related comorbidities in order to determine the risk of CFS following persistent DES.

Previous studies have typically used a retrospective study design to interpret the association between DES and CFS [39, 40]. However, we used a prospective, national-wide, population-based cohort study to investigate the incidence of CFS and the risk of developing CFS among DES patients. We also considered whether CFS-related symptoms and comorbidities, such as immune dysregulation, overlapped [41–44]. The results showed that patients with any comorbidity had a higher risk of developing CFS. In addition, having more frequent medical visits and more severe DES showed a gradient-like association with developing CFS. However, the unadjusted HR indicated a slightly elevated risk among both the DES and non-DES cohorts. In addition, the presence of an association between DES and CFS also indicated an elevated risk. For example, the prevalence of SS was associated with symptoms of DES, and DES, as a surrogate marker, may have resulted in a slightly elevated risk of CFS. The current diagnostic criteria are majority based on the presence of signs and symptoms than on etiology, mainly due to the fact that a fully explanation of the association between etiological factors and pathological mechanisms has not yet been carried out. Therefore, interventions aimed at the management of these hidden markers and at their implications for clinical practice are clearly warranted.

Previous studies have also observed an association between CFS and relevant comorbidities and have demonstrated that the causal relationship between CFS and these comorbidities is difficult to dismantle [30, 31, 45–48]. We hypothesized that having more comorbidities would increase the risk for other chronic diseases, such as persistent fatigue. Moreover, the pathogenesis of DES was partially attributable to SS, which would account for immune dysregulation [33]. Thus, we proposed that DES would be reflected in the immune dysregulation phenomenon and complicated by relative chronic diseases and that CFS would be one possible complication. Therefore, we conducted this prospective cohort study to clarify the risk of developing CFS and associated comorbidities among patients with DES. Moreover, the various time period would be existed different subsequent risk of CFS on coming status using this present approach as time spiral model [49]. Although we used a prospective cohort study design to examine data from the NHIRD in Taiwan, this study has several weaknesses. For example, the Taiwanese NHIRD lacks data at the individual level, but given that a diagnosis of CFS is based on self-reported symptoms and the criteria described above, we were able to rule out any other diseases even though CFS was previously disregarded in Taiwan. In addition, using the NHIRD to assess the risk of CFS resulted in a greater accuracy but a lower effect size. Furthermore, SS and antidepressant use were the major risk factors contributing to DES, thus we tried to adjust for these comorbidities to avoid confounding as much as possible. Moreover, overlapping self-reported symptom disorders may have interfered with the risk assessment of CFS. For example, a previous study was shown that FM is diagnosed more often among SLE patients than among SS patients [50]. This study also showed that FM seems to contribute to constitutional symptoms more often among SLE patients than among SS patients suggesting a differential cause of fatigue and widespread pain in these two different connective tissue diseases. Thus, CFS may contribute to an independent underlying etiological factor that is also linked to DES. Our findings showed that the risk of developing CFS is associated with DES as well as existing comorbidities. In addition, the onset of DES was correlated with the incidence of CFS later in life; therefore, DES could serve as a surrogate marker in clinics and assistant in improving the diagnosis of CFS.

## MATERIALS AND METHODS

### Data source

The present study examined the hypothesis that DES is a risk factor for developing CFS. To examine this hypothesis, we used a population-based prospective cohort study design with selected datasets derived from the NHIRD, which is provided to investigators in Taiwan for research purposes. The National Health Insurance Program started in 1995 and has enrolled more than 98% of the residents in Taiwan. Furthermore, the NHIRD had accumulated a decade of data as previously described [25, 26].

Several datasets were combined for this analysis. NHIRD experts constructed specific data subsets from the NHIRD registration files and original claim data. This study utilized the Longitudinal Health Insurance Dataset 2000, which consists of 1,000,000 randomly selected enrollees sampled from the Registry for Beneficiaries in the year 2000 and following its related accuracy of diagnoses and validation representation in NHIRD as previous descripted [27, 28]. And there were several diseases which were identified and confirmed in Registry for catastrophic illness patients (HV) data files. This study was approval by the Institutional Review Board of the China Medical University Hospital (CMUH104-REC2-115-CR2).

### Identification of study cohorts

The DES cohort of the present study included ambulatory patients with DES (ICD-9-CM code, 370.33) who were aged 20 years or older, and newly diagnosed with DES at the interval (2005–2007). Patients with pre-existing CFS at baseline were excluded. The control group (non-DES cohort) included patients diagnosed with CFS (ICD-9-CM 780.71) from the starting of interval to December 31, 2011 who did not have DES syndrome. Patients were excluded if they were younger than 20 years old or had missing data (null value). The DES and non-DES cohort were frequency-matched by age (5-year interval), gender, and first claim index year. Each study subject was followed until CFS was diagnosed or the patient was censored because of death, loss to follow-up, withdrawal from the database, or until the end of 2011.

### Additional variables of interest

Age was analyzed as a categorical variable (20–64 years old and 65 or older) and as a continuous variable. Baseline comorbidities included any cancer (ICD-9-CM 140–208 from HV), systemic lupus erythematosus (SLE) (ICD-9-CM 710.0 from HV), SS (ICD-9-CM 710.2 from HV), rheumatoid arthritis (RA) (ICD-9-CM 714 from HV), depression (ICD-9-CM 296.2x–296.3x, 300.4, 311.x), anxiety (ICD-9-CM 300.0, 300.2, 300.3, 308.3, and 309.81), sleep disturbances (ICD-9-CM 307, 327, and 780.5), irritable bowel syndrome (IBS) (ICD-9-CM 564.1), and FM (ICD-9-CM 729.1).

### Statistical analyses

Comparisons between patients with DES and patients without DES were conducted using the test for nominal variables and the Student’s *t*-test for continuous variables. The cumulative incidence of CFS was estimated for the two cohorts using the Kaplan-Meier method, and the log-rank test was applied to examine the differences between the two groups. We calculated the age-, sex-, and comorbidity-specific incidence density rates of CFS per person-years in DES and non-DES, separately. We applied univariate and multivariate Cox proportional hazard models to assess the hazard ratios (HRs) with 95% confidence intervals (CIs) for developing CFS among DES patients and non-DES patients. The multivariate Cox models were adjusted for age, sex, and comorbidities, and following assessed adjusted hazard ratios (aHRs). Data management and statistical analyses were performed with SAS 9.4 (SAS Institute, Cary, NC, USA). Statistical significance was defined as *p <* 0.05 (two-sided).

## CONCLUSIONS

In this clinical observation study, the relative risk for CFS between DES and its relative health cohorts was potentially causally associated with more CFS related morbidities and correlated to the risk elevation. If this finding was replicated in Taiwan Biobank or a community-based cohort, these findings may have implications for counseling and managing uncertainty DES-related morbidities to avoid incident CFS in Taiwan.
